# Optimal duration of *ex vivo* lung perfusion for heat stress-mediated therapeutic reconditioning of damaged rat donor lungs

**DOI:** 10.1093/ejcts/ezaf027

**Published:** 2025-01-31

**Authors:** Roumen Parapanov, Anne Debonneville, Manon Allouche, Jérôme Lugrin, Tanguy Lugon-Moulin, Etienne Abdelnour-Berchtold, Lucas Liaudet, Thorsten Krueger

**Affiliations:** The Services of Thoracic Surgery, University Hospital, Lausanne, Switzerland; Adult Intensive Care Medicine, University Hospital, Lausanne, Switzerland; The Services of Thoracic Surgery, University Hospital, Lausanne, Switzerland; Adult Intensive Care Medicine, University Hospital, Lausanne, Switzerland; The Services of Thoracic Surgery, University Hospital, Lausanne, Switzerland; The Services of Thoracic Surgery, University Hospital, Lausanne, Switzerland; Adult Intensive Care Medicine, University Hospital, Lausanne, Switzerland; The Services of Thoracic Surgery, University Hospital, Lausanne, Switzerland; The Services of Thoracic Surgery, University Hospital, Lausanne, Switzerland; Adult Intensive Care Medicine, University Hospital, Lausanne, Switzerland; The Services of Thoracic Surgery, University Hospital, Lausanne, Switzerland

**Keywords:** *Ex vivo* lung perfusion, Heat shock response, Heat stress reconditioning, Animal model, Warm ischaemia

## Abstract

**OBJECTIVES:**

Transient heat stress (HS) application during experimental *ex vivo* lung perfusion (EVLP) of warm ischaemic (WI) rat lungs produces a range of therapeutic benefits. Here, we explored whether different EVLP durations after HS application would influence its therapeutic effects.

**METHODS:**

In protocol 1, WI rat lungs were exposed to HS (41.5°C, 60–90 min EVLP), and EVLP was maintained for 3, 4.5 or 6 h (*n* = 5/group), followed by physiological measurements (compliance, oedema, oxygenation capacity). In protocol 2, WI rat lungs treated with (HS groups) or without HS (control groups) were maintained for 3 or 4.5 h EVLP (*n* = 5/group), followed by physiological evaluation and measurements (lung tissue) of heat shock proteins (HSP70, HSP27, HS90, GRP78), endogenous proteins (surfactant protein-D, CC16, platelet endothelial cell adhesion molecule-1), anti-apoptotic (Bcl2, Bcl-xL) and pro-apoptotic proteins (Bcl2-associated X protein, CCAAT/enhancer binding-protein homologous protein), antioxidant enzymes (heme-oxygenase-1, nicotinamide di-phospho-nucleotide dehydrogenase quinone-1) and nitrotyrosine (oxidative stress biomarker).

**RESULTS:**

In protocol 1, physiological variables were stable after 3 and 4.5 h but deteriorated after 6 h. In protocol 2, at 3 h EVLP, HS-treated lungs differed from controls by higher expression of HSP70 and heme-oxygenase-1, and lower CC16 expression. In contrast, at 4.5 h EVLP, HS-treated lungs displayed improved physiology, higher levels of all HSPs, preserved or increased expression of surfactant protein-D, CC-16 and platelet endothelial cell adhesion molecule-1, increased antioxidant and anti-apoptotic proteins, and reduced pro-apoptotic proteins and nitrotyrosine.

**CONCLUSIONS:**

The protective effects of HS application during EVLP of WI-damaged rat lungs strictly depend on the duration of post-HS recovery. An EVLP duration of 4.5 h appears to optimize the therapeutic potential of HS, while maintaining lungs in a stable physiological state.

## Graphical abstract

**Figure:**
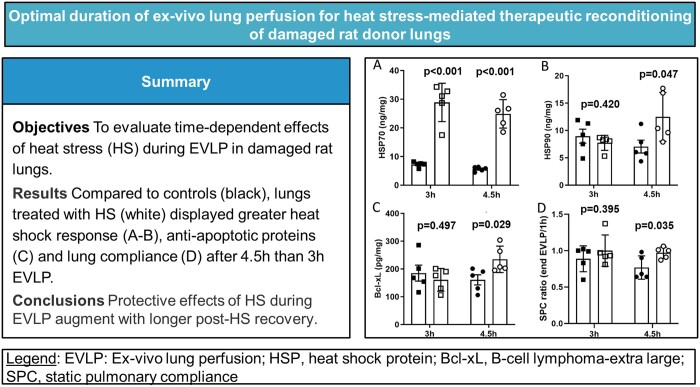

## INTRODUCTION

Lung transplantation (LTx) is the unique treatment for end-stage lung diseases, but it is limited by the shortage of transplantable lungs. To cope with this problem, many lung transplant centres implemented *ex vivo* lung perfusion (EVLP) for the physiological evaluation of marginal donor lungs [1, 2]. EVLP may also allow the administration of therapies to improve lung function and reduce the risk of primary graft dysfunction after LTx, as demonstrated in experimental studies [3, 4]. Beyond pharmacological treatments, EVLP could also serve to stimulate endogenous protective responses following the *ex vivo* application of a physiological stress. One such stimulus can be realized by exposing lungs to a transient non-lethal mild heat stress (HS), which confers resistance to future stressful stimuli by triggering a heat shock response through the induction of heat shock proteins (HSPs) acting as molecular chaperones [5]. In this respect, we recently demonstrated that an *ex vivo* mild HS application to damaged rat lungs reduced cellular injury and physiological deterioration after EVLP and LTx [6, 7].

The molecular signals triggered by HS evolve according to certain chronological steps. During initiation of the HS response, there is a global repression of protein synthesis accompanied by the selective expression of HSPs [8]. The return to normothermic conditions after HS allows the restoration of cell homeostasis and the *de novo* expression of genes necessary for stress adaptation [5]. The kinetic expression of heat-responsive genes indicates that post-HS recovery time is essential for the resistance conferred by this strategy. Accordingly, understanding such kinetics appears crucial to optimize the therapeutic benefits of HS preconditioning [9]. Therefore, this study aimed to investigate the influence of various recovery times after HS application during EVLP on the activation of endogenous protective mechanisms to determine the best time window for HS preconditioning during EVLP.

## MATERIAL AND METHODS

### Animals

All experiments were approved by our local animal committee (Direction générale de l’agriculture, la viticulture et affaires vétérinaires de l’Etat de Vaud, authorizations Nr 3456, 21 April 2019, and Nr 3456x1, 29 October 2022). Twenty-five male Sprague-Dawley rats (350–450 g, Charles River, Saint-Germain-Nuelles, France) were used in this study and treated in accordance with ‘Guidelines for the Care and Use of Laboratory Animals’ (NIH Publication no. 96-23).

### Experimental model

We used our published model of warm ischaemic rat lungs and EVLP, as previously described [6, 7]. Briefly, anaesthetized, tracheotomized and mechanically ventilated rats were sacrificed after systemic anticoagulation (intravenous heparin 600 IU) and kept *in situ* for 1 h at room temperature (warm ischaemic time). The pulmonary artery and left ventricle were then cannulated via a median sternotomy, lungs were flushed (PERFADEX^®^ Plus, 4°C, 25 ml) and inflated (7 ml/kg, FiO_2_ 0.5). The heart-lung block was removed, stored at 4°C in PERFADEX^®^ Plus for 1 h (cold ischaemia), weighted and mounted on the EVLP system.

### *Ex vivo* lung perfusion and heat stress preconditioning

EVLP and HS were performed as previously described [6]. Briefly, the perfusate (Steen^®^ solution) was progressively warmed from 16° to 37° over 1 h, at increasing flow (from 2 to 7.5% estimated cardiac output). Ventilation (Flexivent FX3 ventilator, SCIREQ Inc., Montréal, Canada) was started at 35°C perfusate temperature (tidal volume 3 ml/kg, rate 7/min, room air), then increased to 6 ml/kg and 30/min at 37°C. HS was applied after 1 h EVLP by rapidly warming the perfusate at 41.5°C for 30 min, followed by return to 37°C until the end of EVLP. The temperature of the perfusate was monitored at the entry of the pulmonary artery with a TES-1303 Type-K digital thermometer (TES Electrical Electronic Corp., Taipei, Taiwan).

### Experimental protocols

**Figure 1:**
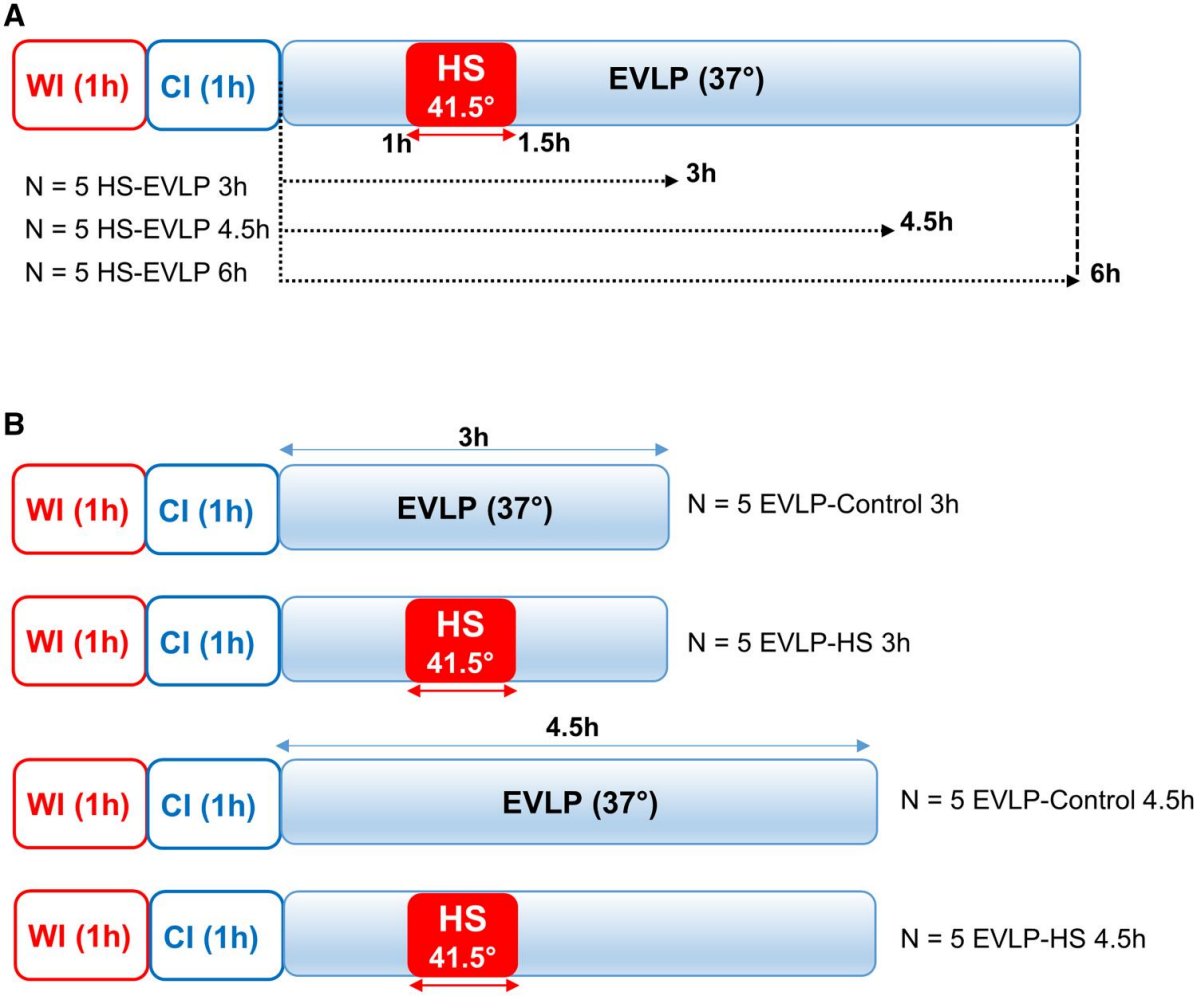
Experimental design. (**A**) Protocol 1: lungs exposed to 1 h warm ischaemia (WI) and 1 h cold ischaemia (CI) were perfused in an *ex vivo* lung perfusion (EVLP) system for 3, 4.5 or 6 h at 37°C, with the application of a transient heat stress (HS) at 41.5° from 1 to 1.5 h EVLP. (**B**) Protocol 2: lungs exposed to 1 h WI and 1 h CI were perfused in an EVLP system for 3 or 4.5 h, with (EVLP-HS) or without (EVLP-control) the application of a transient HS at 41.5° from 1 to 1.5 h EVLP.

The experimental design is depicted in Fig. 1. In protocol 1, 3 groups of lungs (*n* = 5/group) were exposed to HS for 30 min after 1 h EVLP as described above (60–90 min EVLP) and then maintained under EVLP for 3 h (HS_**3h**_), 4.5 h (HS_**4.5h**_) or 6 h (HS_**6h**_) to allow, respectively, 90, 180 and 270 min recovery time after HS. In protocol 2, we compared lungs exposed to HS and perfused 3 h (HS_3h_, *n* = 5) or 4.5 h (HS_4.5h_, *n* = 5) to parallel control groups that did not undergo HS (Ctrl_**3h**_, *n* = 5 and Ctrl_**4.5h**_, *n* = 5). At the end of EVLP, the heart-lung block was weighed and the difference from weight before EVLP was used as an index of lung oedema, then the lungs were kept frozen at −80°C for further analyses. In both protocols, groups were alternate each day but were not randomized. The sample size was estimated from our previous study on HS-preconditioning [7], in which we measured several biomarkers in the perfusate at selected time-points during EVLP. At the 4.5 h time-point, biomarkers differed by a mean proportion of 1.84 between HS and control groups, with a mean standard deviation of 47%. Assuming comparable proportions and variance in the present study, we calculated (α: 5%, power: 80%, two-sided test) that a size of *n* = 5/group (HS and controls) would be appropriate for meaningful analyses.

### Measurements

#### Physiological measurements

Static pulmonary compliance and peak airway pressure (Pmax) were determined as described previously [6, 7], expressed as the ratio of the value at the end of EVLP to that at 1 h EVLP (before HS application) [6, 7]. The partial pressure of O_2_ was measured at the end of EVLP in the effluent perfusate (CG4+ cartridge, Abbott i-STAT analyzer, East Windsor, KJ).

#### Biological measurements

##### Heat shock protein expression

Lung tissue was homogenized in RIPA buffer with proteases/phosphatase inhibitors and sonicated. The concentrations of HSPs (HSP70, HSP27, HSP90 and HSPA5/GRP78) were determined using enzyme-linked immunosorbent assays (Supplementary Material, Table S1), expressed in ng/mg tissue protein, measured by the BCA assay (Thermo Scientific Pierce, Rockford, IL, USA).

##### Expression of lung epithelial and endothelial proteins, pro- and anti-apoptotic proteins, antioxidant proteins and 3-nitrotyrosine

Lung homogenates were assayed using specific enzyme-linked immunosorbent assays (Supplementary Material, Table S1) for the epithelial proteins Surfactant protein-D (SP-D) and Clara cell protein (CC16), the endothelial protein platelet endothelial cell adhesion molecule-1 (PECAM-1), the anti-apoptotic proteins B-cell CLL/lymphoma-2 (Bcl2) and Bcl2-like protein-1 (Bcl-xL), the pro-apoptotic proteins Bcl2-associated X protein (Bax), CCAAT/enhancer binding-protein (C/EBP) homologous protein (CHOP), as well as the antioxidant proteins NAD(P)H quinone-1-dehydrogenase (NQO-1) and heme oxygenase-1 (HO-1). The levels of lung 3-nitrotyrosine (3-NT) were determined as an index of nitroxidative stress. All data were expressed in ng/mg or pg/mg tissue protein.

### Statistical analysis

Results are presented as means±SD. In protocol 1, measurements between the 3 groups were compared using one-way ANOVA followed by Tukey test. In protocol 2, comparisons between HS groups and their respective controls at 3 h and 4.5 h were done by unpaired two-tailed *t-*test after log transformation of the data in case of non-normal distribution (assessed by the Shapiro–Wilk test). Furthermore, we compared relative protein expressions (fold changes) between the HS groups and their respective controls at 3 h and 4.5 h EVLP, using unpaired two-tailed *t-*test. Statistical significance was assigned to a *P* < 0.05. GraphPad Prism, version 10.1.2 (GraphPad Software Inc., La Jolla, CA) was used for all statistical analyses.

## RESULTS

### First experimental protocol: comparison of functional outcome of lungs exposed to heat stress and different recovery times

**Figure 2:**
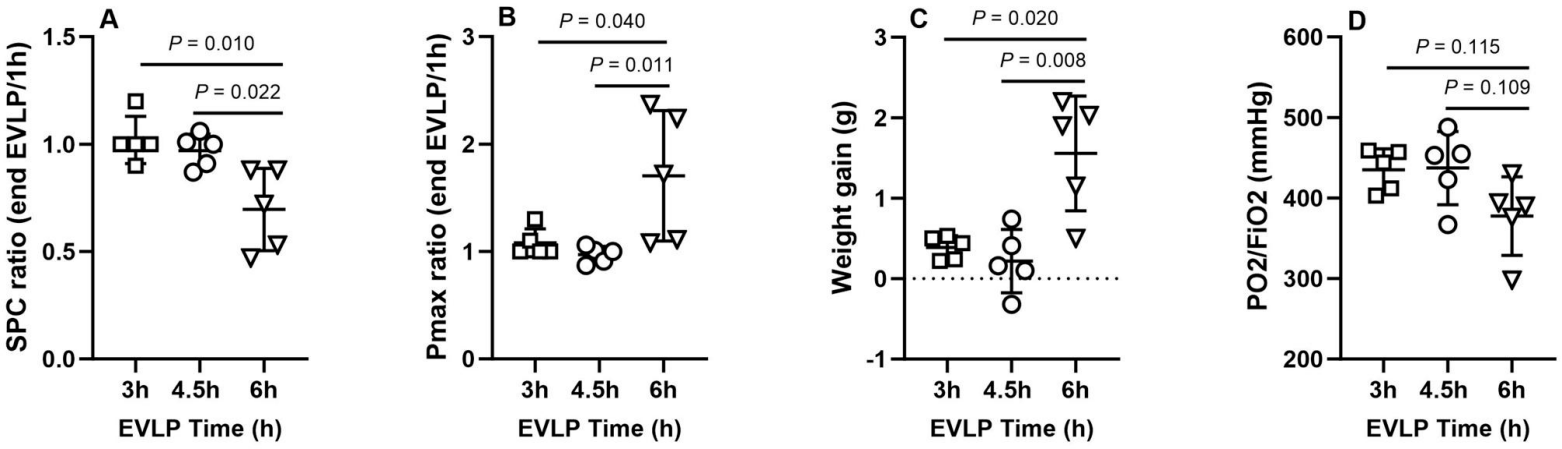
Pulmonary physiological variables following heat stress (HS) and *ex vivo* lung perfusion (EVLP) durations of 3 h, 4.5 h and 6 h. (**A**) Static pulmonary compliance (SPC), expressed as the ratio of SPC at the end of EVLP/1 h EVLP. (**B**) Maximal airway pressure (Pmax) expressed as the ratio of Pmax at the end of EVLP/1h. (**C**) Lung oedema development, assessed by the weight gain of the lungs at the end of EVLP. (**D**) Lung oxygenation capacity expressed in P/F ratio. *N* = 5 groups

As indicated in Fig. 2, lungs from the group HS_**6h**_ displayed significant reduction of static pulmonary compliance (Fig. 2A) and increase in Pmax (Fig. 2B), associated with greater oedema development (weight gain during EVLP, Fig. 2C) and a non-significant trend for reduced oxygenation capacity (P/FO_2_ in the EVLP effluent, Fig. 2D). Owing to this significant deterioration of lungs from the HS_**6h**_ group, further investigations were performed only in the groups HS_**3h**_ and HS_**4.5h**_ and their respective controls_**.**_

### Second experimental protocol: comparison of lungs exposed or not to heat stress after 3 h and 4.5 h *ex vivo* lung perfusion

#### Physiological variables

**Figure 3:**
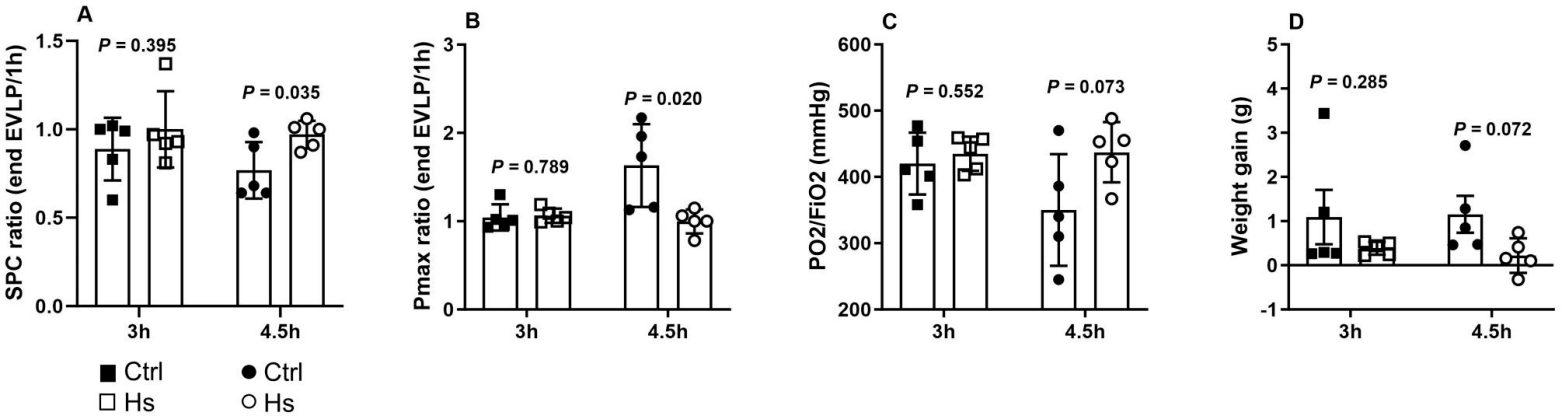
Lung physiological variables after 3 and 4.5 h *ex vivo* lung perfusion (EVLP). (**A**) Static pulmonary compliance (SPC), expressed as SPC ratio at the end of EVLP/1 h EVLP. (**B**) Maximal airway pressure (Pmax) expressed as the ratio of Pmax at the end of EVLP/1 h. (**C**) Lung oxygenation capacity expressed in P/F ratio. (**D**) Lung oedema development, assessed by the weight gain of the lungs at the end of EVLP. *N* = 5 groups.

After 3 h EVLP, HS-treated lungs (HS_**3h**_) showed similar respiratory parameters compared to Ctrl_**3h**_ lungs (Fig. 3A–D). In contrast, after 4.5 h EVLP, lungs from the HS_**4.5h**_ group demonstrated significantly better functional outcomes than lungs from the Ctrl_**4.5h**_ group, with significantly higher static pulmonary compliance, lower Pmax and a trend (*P* = 0.07) for better oxygenation and reduced oedema (Fig. 3A–D).

#### Biological variables

##### Heat shock proteins

**Figure 4:**
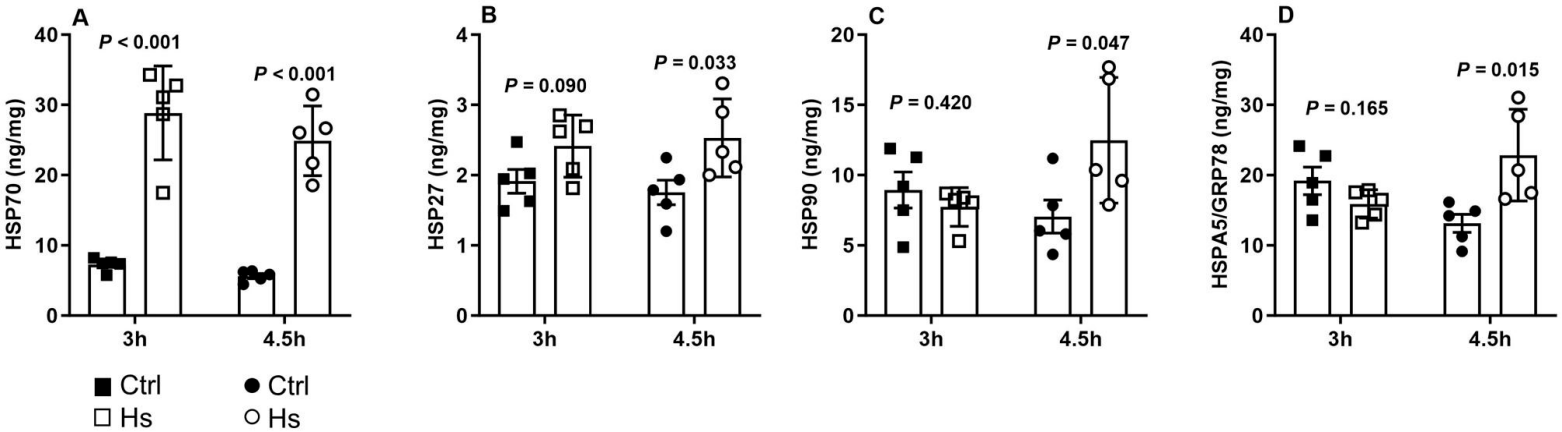
Lung expression of heat shock proteins at the end of 3 or 4.5 h *ex vivo* lung perfusion (EVLP). Lung tissue concentrations of (**A**) HSP70, (**B**) HSP27, (**C**) HSP90 and (**D**) HSPA5/GRP78. *N* = 5 groups.

As shown in Fig. 4A, a significant induction of HSP70 occurred in HS_**3h**_ and HS_**4.5h**_ compared to their respective controls. In contrast, levels of HSP27 (Fig. 4B), HSP 90 (Fig. 4C) and HSPA5/GRP78 (Fig. 4D) did not differ between HS_**3h**_ and Ctrl_**3h**_, whereas they were significantly increased in HS_**4.5h**_ compared to Ctrl_**4.5h**_. When expressed as fold change in comparison to controls (not shown), the change tended to be greater for HSP70 and HSP27 and was significantly greater for HSP90 and HSPA5/GRP78 at 4.5 h than at 3 h.

##### Lung epithelial and endothelial proteins

**Figure 5:**
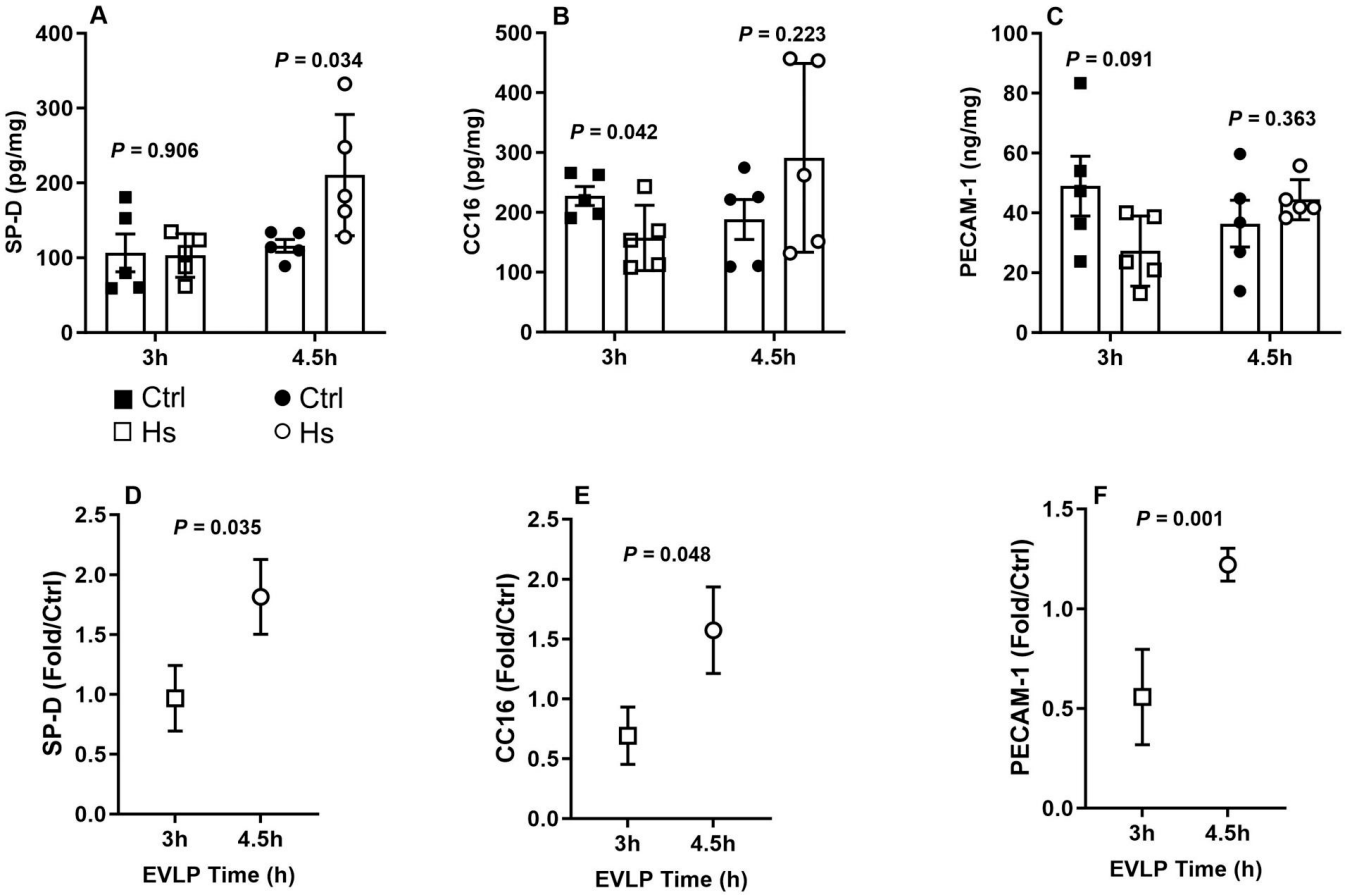
Expression of lung endogenous proteins at the end of 3 h or 4.5 h *ex vivo* lung perfusion (EVLP). (**A**) Surfactant protein D (SP-D), (**B**) Clara cell protein (CC16), (**C**) Platelet-endothelial cell adhesion molecule-1 (PECAM-1), all expressed in ng/mg or pg/mg lung tissue protein. (**D**) SP-D, (**E**) CC16, (**F**) PECAM-1, expressed as fold change in heat stress (HS) versus controls at each time-point. *N* = 5 groups.

Lungs in both Ctrl_**3h**_ and HS_**3h**_ groups displayed similar expression of SP-D (Fig. 5A), whereas expression of CC16 and PECAM-1 decreased in HS_**3h**_ compared to Ctrl_**3h**_ (Fig. 5B and C). At 4.5 h EVLP, a significant increase of SP-D expression occurred in HS_**4.5h**_ group (Fig. 5A), while CC16 and PECAM-1 did not significantly vary with respect to Ctrl_**4.5h**_ (Fig. 5B and C). The variation of protein expression in the HS groups compared to their respective controls (fold changes) indicated that the changes were significantly greater for all examined proteins after 4.5 h than after 3 h EVLP (Fig. 5D–F).

##### Anti- and pro-apoptotic proteins

**Figure 6:**
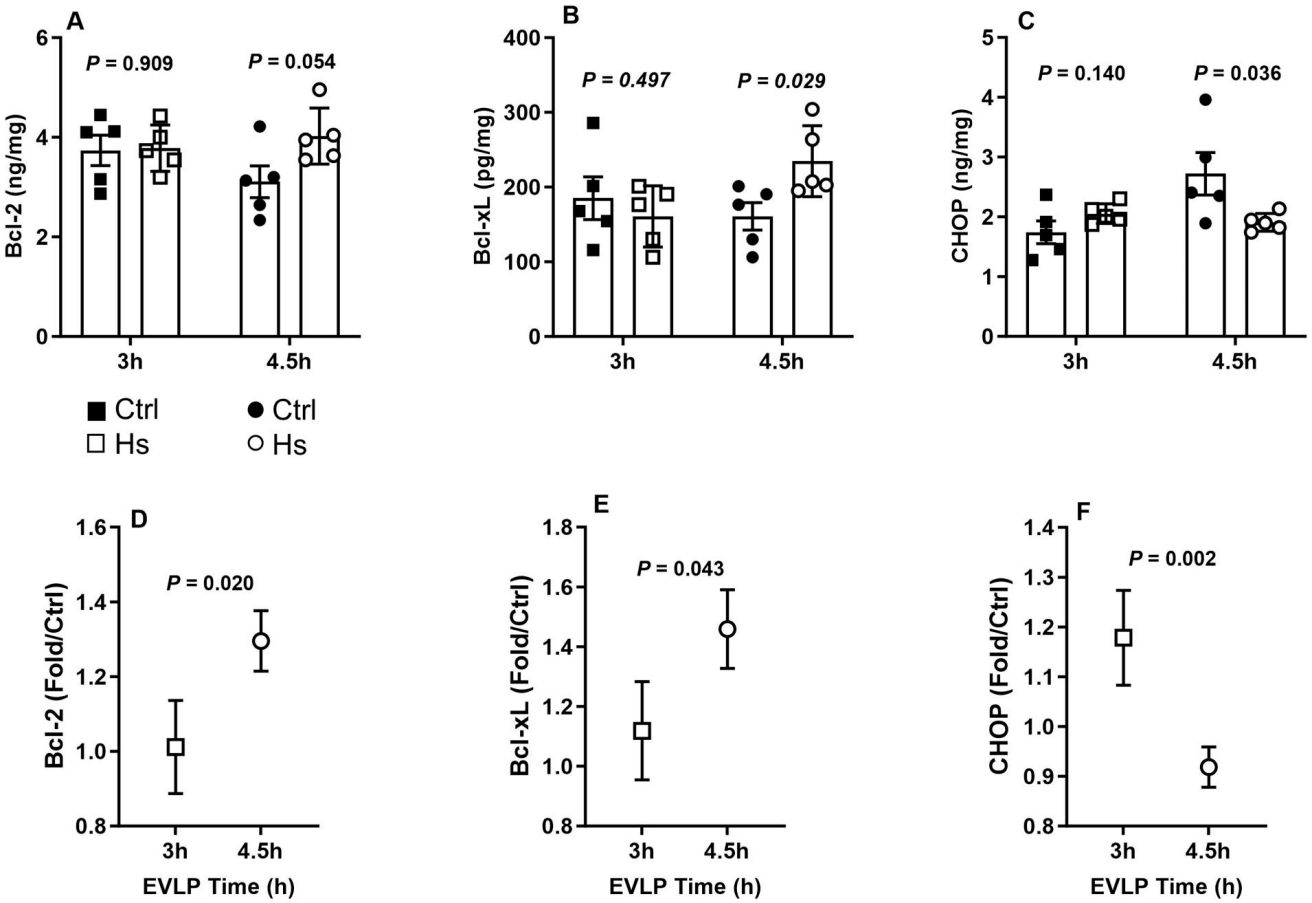
Pro- and anti-apoptotic proteins in lung tissue at 3 and 4.5 h *ex vivo* lung perfusion (EVLP). (**A**) B-cell leukemia/lymphoma-2-protein (Bcl-2), (**B**) B-cell lymphoma-extra large (Bcl-xL), (**C**) CCAAT/enhancer binding-protein (C/EBP) homologous protein (CHOP), expressed in ng/mg or pg/mg lung protein. Relative expression (fold changes in heat stress (HS) versus control lungs at 3 and 4.5 h EVLP) of (**D**) Bcl-2, (**E**) Bcl-xL, (**F**) CHOP. *N* = 5 groups.

After 3 h EVLP, the levels of anti-apoptotic Bcl-2 and Bcl-xL (Fig. 6A and B) and pro-apoptotic CHOP (Fig. 6C) and Bax (not shown) proteins were not significantly influenced by HS. In contrast, after 4.5 h EVLP, lungs from the HS_**4.5h**_ group exhibited significantly higher levels of Bcl-2 and Bcl-xL, lower levels of CHOP (Fig. 6A–C) and a trend for lower Bax levels (not shown) compared to the Ctrl_**4.5h**_ group. This was further illustrated by the relative variations of the different proteins, showing that the influence of HS was significantly more pronounced after 4.5 h than 3 h EVLP, with a positive impact on anti-apoptotic Bcl-2 and Bcl-xL (Fig. 6D and E), and a negative impact on pro-apoptotic CHOP (Fig. 6F).

##### Antioxidant proteins and 3-nitrotyrosine

**Figure 7:**
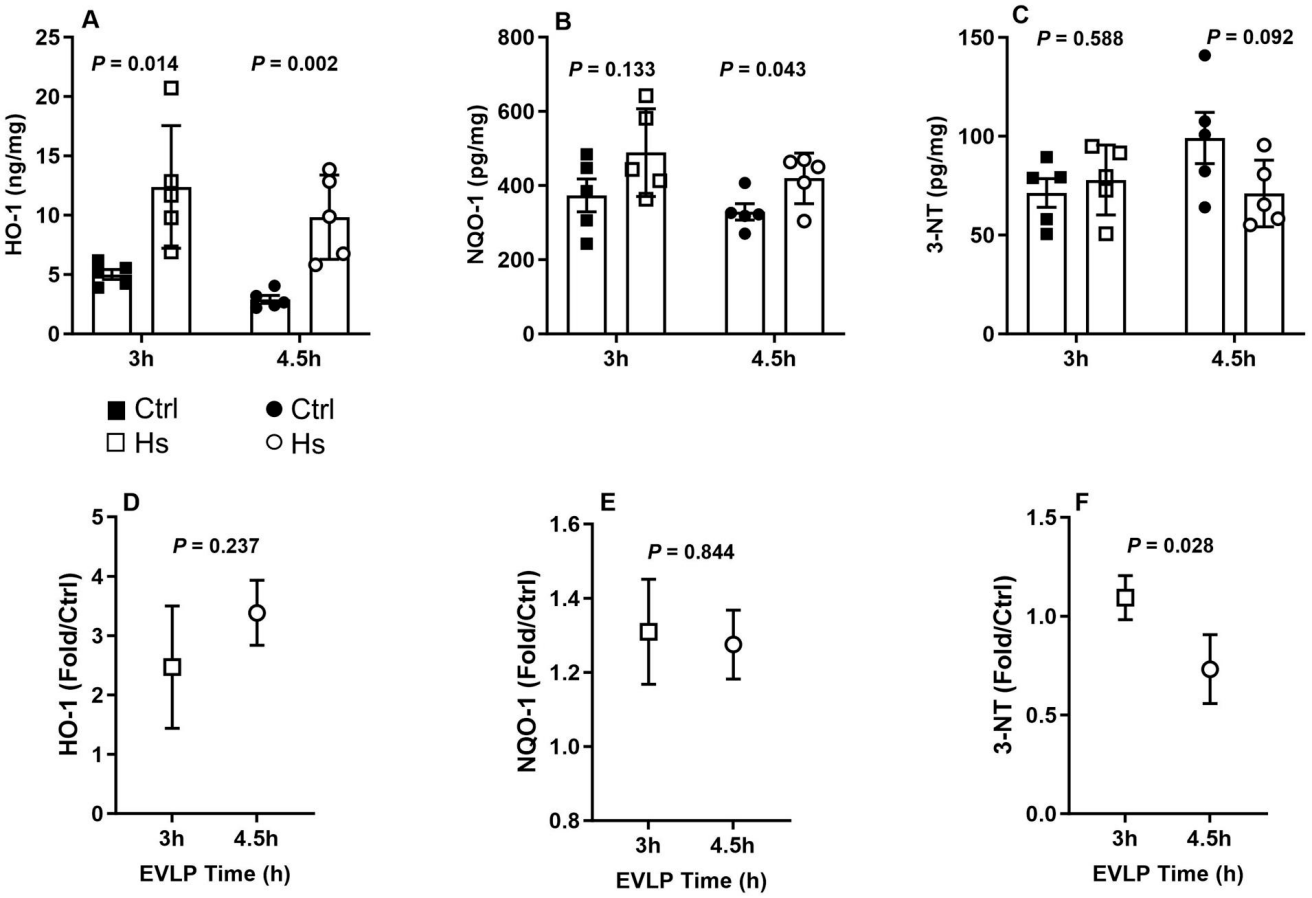
Antioxidant proteins and 3-nitrotyrosine in lungs at the end of *ex vivo* lung perfusion (EVLP) for 3 or 4.5 h. Lung tissue levels of (**A**) HO-1 (heme oxygenase-1), (**B**) NQO-1 [NAD(P)H quinone dehydrogenase 1], (**C**) 3-NT (3-nitrotyrosine). Relative expression [fold changes heat stress (HS) versus controls] of (**D**) HO-1, (**E**) NQO-1 and (**F**) 3-NT at 3 and 4.5 h EVLP. *N* = 5 groups.

While both HS_**3h**_ and HS_**4.5h**_ groups displayed a significantly increased expression of HO-1 in comparison to their respective controls (Fig. 7A), a significant increase of NQO-1 only occurred in HS_**4.5h**_ group (Fig. 7B). Levels of 3-NT were comparable between lungs from HS_**3h**_ and Ctrl_**3h**_ groups, whereas they tended to be lower in HS_**4.5h**_ compared to Ctrl_**4.5h**_ (Fig. 7C). Fold change analyses revealed a comparable influence of HS on HO-1 and NQO-1 after 3 and 4.5 h EVLP (Fig. 7D and E). In contrast, HS had a significantly larger effect on 3-NT after 4.5 h than 3 h EVLP (Fig. 7F).

## DISCUSSION

We recently demonstrated that the *ex vivo* application of a transient mild HS to damaged donor lungs reduced lung injury and dysfunction during EVLP and LTx, via the expression of HSPs and reduced inflammation, oxidative stress and cell death [6, 7]. Owing to the dynamic nature of the HS response [9], we postulated that different post-HS recovery times might influence its therapeutic potential. In a first set of experiments, we observed that lungs subjected to HS and maintained under EVLP for 3 and 4.5 h exhibited stable physiology, contrasting with reduced compliance, increased airway pressure and oedema formation after 6 h. We found in a previous study [7] that HS-treated lungs were protected against endothelial damage and dysfunction after 6 h EVLP, despite a slight degree of oedema and reduced compliance, in agreement with our present findings. Therefore, even though lungs remain significantly protected by HS after 6 h EVLP, they may still develop moderate physiological deterioration, which indicates that the protective effects of HS cannot be fully maintained for extended (6 h) durations of EVLP. Accordingly, we decided to restrict our subsequent investigations to EVLP durations of 3 h and 4.5 h, during which HS-treated lungs were reperfused without any apparent functional impairment.

The hallmark of the HS response is the induction of various HSPs [5]. Previous studies indicated that the main stress-inducible HSP70 and HSP27 are rapidly expressed (30 min–2h) after HS and then remain elevated for several hours [7, 9]. In agreement with these findings, we found that the induction of these 2 HSPs was comparable after 3 and 4.5 h, implying that extending post-HS recovery time beyond 90 min (3 h EVLP) is not necessary to obtain their full expression. In contrast, 2 additional inducible HSPs (HSP90 and HSPA5/GRP78) were significantly expressed only after 4.5 h, indicating that an EVLP model allowing 180 min post-HS recovery (EVLP 4.5 h) is necessary to optimize the expression of major inducible HSPs in physiologically intact lungs. This is an important finding, given that the efficiency of the protective functions of HSPs directly correlates with their expression levels [5].

An initial temporary downregulation of general protein transcription and translation is a characteristic response to HS, enabling the reduction of cellular misfolded protein accumulation [6, 8]. Consistently, we found that the expression of key epithelial (SP-D, CC16) and endothelial (PECAM-1) proteins was differentially influenced according to the duration of post-HS recovery. SP-D, synthesized by alveolar type II cells, plays important roles in lung innate immune defences [10], whereas CC16, secreted by Clara Cells, has potent antioxidant/anti-inflammatory roles [11]. Notably, the interest of SP-D and CC16 as biomarkers of epithelial injury and primary graft dysfunction after LTx has been highlighted by previous investigators [12, 13]. PECAM-1 is an endothelial protein essential for endothelial barrier integrity [14]. When comparing the relative expression of SP-D, CC16 and PECAM-1 in HS and control lungs, we found that it was either unchanged or reduced after 3 h but was increased after 4.5 h, consistent with an early slowdown of transcription followed by recovery. We conclude, therefore, that an EVLP duration of 4.5 h, allowing sufficient post-HS recovery time, is required to secure the appropriate expression of key pulmonary homeostatic proteins.

Apoptosis represents an important mode of cell death in lung ischaemia–reperfusion and LTx, and lung grafts undergoing warm ischaemia display a pro-apoptotic transcriptomic signature [15] and suffer enhanced apoptosis during EVLP [16]. Intrinsic (mitochondrial) apoptosis is regulated by the balanced expression of anti (Bcl-2, BcL-xL) and pro-apoptotic (Bax) proteins [17], and several HSPs can shift this balance towards a predominantly anti-apoptotic phenotype [18, 19]. Clinically, the role of several genes of the Bcl-2 family as potential biomarkers and targets of treatment for primary graft dysfunction has been recently underscored [20]. We found that HS did not alter the expression of these proteins after 3 h EVLP, contrasting with a significant enhancement of Bcl2 and Bcl-XL expression after 4.5 h. This was associated with decreased expression of CHOP, a pro-apoptotic protein induced upon endoplasmic reticulum stress [21], an effect likely related to the strong induction of HSPA5/GRP78 at 4.5 h EVLP, a regulator of endoplasmic reticulum homeostasis preventing CHOP induction and downstream apoptosis [21]. Taken together, these data indicate that extending post-HS recovery time from 90 (EVLP 3 h) to 180 min (EVLP 4.5 h) triggered important pro-survival signals in reperfused lungs.

A consistent response to HS is a steady protection against oxidative stress, largely mediated by the activation of the NRF-2 transcription factor and downstream targets such as HO-1 and NQO-1 [22, 23]. Accordingly, the expression of these 2 antioxidant proteins was promoted in lungs exposed to HS to a comparable extent after 3 or 4.5 h EVLP. Therefore, such adaptation appears as an early response to HS with no apparent time-dependent amplification. Nevertheless, we found that the levels of 3-NT, a footprint of the strong oxidant peroxynitrite [24], were attenuated after 4.5 h, but not 3 h EVLP. This suggests that a longer post-HS recovery time may favour more extensive protection against oxidative stress, and therefore, that an EVLP protocol of 4.5 h is better suited for optimal antioxidant defences.

### Limitations

Our study has several limitations. First, we did not assess the influence of different post-HS recovery times during EVLP after transplantation. The primary aim of our study was to address the chronology of events set in motion by HS application during the *ex vivo* procedure. Future experiments will be needed to determine whether the time-dependent effects of HS *ex vivo* translate into different outcomes after LTx for an in-depth evaluation of this topic. Second, we detailed the effects of HS at only 2 distinct time-points (3 and 4.5 h). Importantly, our objective was to determine the duration of EVLP after HS application that would maintain the lungs in a functionally intact state while ensuring an optimized expression of HSPs. Such conditions were better fulfilled after 4.5 h than after 3 h EVLP, while after 6 h, the protective effects of HS could not be fully maintained. However, even though the 4.5 h EVLP provided superior outcomes when compared to 3 and 6 h, we cannot exclude that any durations of EVLP within the 3–6-h range could have provided optimal performance.

In summary, our study presents the first evidence of the critical role of post-stress recovery time in the context of therapeutic heat stress for *ex vivo* reconditioning of damaged lungs. Thus, when applying a transient HS after 1 h EVLP, we could demonstrate that a post-HS recovery time of 180 min (4.5 h EVLP) provides a larger benefit than 3 h or 6 h EVLP in terms of HSP induction, expression of endogenous homeostatic proteins and the induction of pro-survival and antioxidant defences, while maintaining the lungs in a state of physiological integrity. Whether a better performance of HS reconditioning could be achieved using different durations of EVLP (between 3 and 6 h) remains to be established. These findings extend the concept of *ex vivo* heat stress application as a non-pharmacological therapy for the rehabilitation and reconditioning of damaged lung grafts.

## Supplementary Material

ezaf027_Supplementary_Data

## Data Availability

All data underlying this article are available in the article and in its online supplementary material.

## Notes

Presented, partly, at the 32nd European Conference on General Thoracic Surgery from the ESTS, Barcelona, Spain, 27 May 2024.

## ABBREVIATIONS

3-NT: 3-nitrotyrosine
Bax: Bcl2-associated X protein
Bcl-2: B-cell leukaemia/lymphoma-2-protein
Bcl-xL: B-cell lymphoma-extra large
CC16: Clara cell secretory protein-16
CHOP: CCAAT/enhancer binding-protein (C/EBP) homologous protein
EVLP: *Ex vivo* lung perfusion
HO-1: Heme-oxygenase-1
HS: Heat stress
HSPs: Heat shock proteins
LTx: Lung transplantation
NQO-1: Nicotinamide di-phospho-nucleotide dehydrogenase quinone-1
PECAM-1: Platelet endothelial cell adhesion-molecule-1
Pmax: Maximal airway pressure
SP-D: Surfactant protein-D
