# Supplementary material for: Optimal duration of ex vivo lung perfusion for heat stress-mediated therapeutic reconditioning of damaged rat donor lungs
Source: Eur J Cardiothorac Surg. 2025 Jan 31;67(2):ezaf027. doi: 10.1093/ejcts/ezaf027 (PMC11831693; doi:10.1093/ejcts/ezaf027)
Supplement: ezaf027_Supplementary_Data [file ezaf027_supplementary_data.docx]

Supplementary Table 1. List of ELISA kits used in the study

| **ELISAs** | **References** | **Supplier** |
| --- | --- | --- |
| Rat HSP70 ELISA kit | ADI-EKS-700B | Enzo Life Sciences |
| Rat HO-1 ELISA kit | ADI-EKS-810A | Enzo Life Sciences |
| Rat HSPB1/HSP27 ELISA kit | LS-F4101 | LifeSpan BioSciences, Inc. |
| Rat HSP90 ELISA kit | LS-F24259 | LifeSpan BioSciences, Inc. |
| Rat HSPA5/GRP78/ Bip ELISA kit | LS-F32597 | LifeSpan BioSciences, Inc. |
| Rat SCGB1A1 / Uteroglobin / CC16 ELISA kit | LS-F12916 | LifeSpan BioSciences, Inc. |
| Rat SFTPD / Surfactant Protein D | LS-F24087 | LifeSpan BioSciences, Inc |
| Rat NQO1/ NAD(P)H dehydrogenase ELISA kit | LS-F32218 | LifeSpan BioSciences, Inc. |
| Rat DDIT3 / CHOP ELISA kit | LS-F39426 | LifeSpan BioSciences, Inc. |
| Rat Bcl-2 like protein 1 (Bcl-xL) ELISA kit | CSB-E13604r | Cusabio Technology LLC |
| Rat B-cell CLL/lymphoma 2 (Bcl2) ELISA Kit | CSB-E08854r | Cusabio Technology LLC |
| Rat Apoptosis Regulator BAX(Bax) ELISA Kit | CSB-EL002573RA | Cusabio Technology LLC |
| Rat 3-Nitrotyrosine (3-NT) ELISA kit | AMS.E02A0670 | Amsbio |
| Rat PECAM-1 | RTFI00178 | Assay Genie |
